# Optical Genome Mapping as a Diagnostic Tool in Pediatric Acute Myeloid Leukemia

**DOI:** 10.3390/cancers14092058

**Published:** 2022-04-19

**Authors:** Julia Suttorp, Jonathan Lukas Lühmann, Yvonne Lisa Behrens, Gudrun Göhring, Doris Steinemann, Dirk Reinhardt, Nils von Neuhoff, Markus Schneider

**Affiliations:** 1 Clinic of Pediatrics III, University Hospital Essen, Virchow-Straße 171, 45147 Essen, Germany; julia.suttorp@uk-essen.de (J.S.); dirk.reinhardt@uk-essen.de (D.R.); nils.vonneuhoff@uk-essen.de (N.v.N.); 2 Department of Human Genetics, Hannover Medical School, 30625 Hannover, Germany; luehmann.jonathan@mh-hannover.de (J.L.L.); behrens.yvonne@mh-hannover.de (Y.L.B.); goehring.gudrun@mh-hannover.de (G.G.); steinemann.doris@mh-hannover.de (D.S.)

**Keywords:** acute myeloid leukemia, cytogenetics, optical genome mapping, MRD monitoring, pediatric

## Abstract

**Simple Summary:**

Treatment of pediatric acute myeloid leukemia (AML) is stratified according to multiple recurrent genetic aberrations, which require for detection of different diagnostic methods such as karyotyping and fluorescence in situ hybridization (FISH). The aim of this study was to analyze whether optical genome mapping (OGM), as a new all-in-one methodological approach, can identify all stratification-relevant genetic aberrations that were described by karyotyping. Therefore, frozen bone marrow and blood cells from 24 pediatric patients with AML, bi-lineage leukemia, and mixed-phenotype acute leukemia collected at diagnosis were analyzed by OGM. The results of OGM were compared with routine diagnostic results from karyotyping and FISH. We show that OGM has much potential to address limitations of cytogenetics and even identify new structural aberrations that can be useful for monitoring minimal residual disease (MRD) in patients without an MRD marker.

**Abstract:**

Pediatric AML is characterized by numerous genetic aberrations (chromosomal translocations, deletions, insertions) impacting its classification for risk of treatment failure. Aberrations are described by classical cytogenetic procedures (karyotyping, FISH), which harbor limitations (low resolution, need for cell cultivation, cost-intensiveness, experienced staff required). Optical Genome Mapping (OGM) is an emerging chip-based DNA technique combining high resolution (~500 bp) with a relatively short turnaround time. Twenty-four pediatric patients with AML, bi-lineage leukemia, and mixed-phenotype acute leukemia were analyzed by OGM, and the results were compared with cytogenetics. Results were discrepant in 17/24 (70%) cases, including 32 previously unknown alterations called by OGM only. One newly detected deletion and two translocations were validated by primer walking, breakpoint-spanning PCR, and DNA sequencing. As an added benefit, in two cases, OGM identified a new minimal residual disease (MRD) marker. Comparing impact on risk stratification in de novo AML, 19/20 (95%) cases had concordant results while only OGM unraveled another high-risk aberration. Thus, OGM considerably expands the methodological spectrum to optimize the diagnosis of pediatric AML via the identification of new aberrations. Results will contribute to a better understanding of leukemogenesis in pediatric AML. In addition, aberrations identified by OGM may provide markers for MRD monitoring.

## 1. Introduction

In children, next to acute lymphoblastic leukemia (ALL), acute myeloid leukemia (AML) is the second most frequently diagnosed blood cancer affecting approximately 120 children per year in Germany [1]. Pediatric AML is a heterogeneous disease exhibiting multiple different recurrent genetic aberrations, including aneuploidies, structural variations (SVs), and gene mutations [2,3]. These aberrations are associated with leukemia’s sensitivity to cytostatic drug treatment and thus form the basis to define AML risk groups (standard-, intermediate-, high-risk group) for the categorization of individual patients and their stratification into subsequent therapy arms. Patients with high-risk mutations receive more intensive therapy than patients with standard-risk mutations. In this background, within the last two decades, the survival probability of children with AML has increased considerably [4], having achieved, as of today, 5-year probabilities for event-free survival (EFS) and overall survival (OS) of 50% ± 2% and 76% ± 4%, respectively [1].

While in the past, treatment intensity was selected merely based on treatment response as defined by a combination of leukocyte count, morphology, and cytogenetic findings, the currently improved probability in OS is mainly due to individualized therapeutic approaches, which are achieved by identification of known mutations harboring relapse risk and subsequent monitoring of minimal residual disease (MRD) [5].

In order to detect the underlying mutations in AML, different diagnostic methods are required comprising karyotyping, FISH, and next-generation sequencing (NGS) of DNA and RNA [6]. Despite the impressive achievements when applying NGS, karyotyping and FISH make up the largest part to detect the high number of aneuploidies and SVs occurring in pediatric AML and thus still form the gold standard in a laboratory specialized in diagnosing AML [6,7,8,9,10]. However, well-known limitations in karyotyping and in FISH comprise a low resolution limited to maximally 4 Mbp and targeted 100 kbp, respectively, the need for cell cultivation techniques, cost-intensiveness, and the necessity of experienced and intensively trained staff.

Optical Genome Mapping (OGM) is a technique that was initially described in 1993 by Schwartz et al. [11] and has recently been offered as a commercially available approach. Ultra-high molecular weight (UHMW) DNA is extracted from fresh or stored frozen BMA or blood specimen and labeled by a non-cutting restriction enzyme at the sequence CTTAAG, which repeats about 15 times every 100 kbp throughout the genome. The specific labeling creates a pattern like a barcode. The DNA strands labeled in this way are stretched and drawn as single ultra-long fragments through nanochannels on a chip, where they are imaged. The resulting images are translated into molecule maps and thus can be compared electronically with the reference genome at a resolution down to 500 bp. By this means, OGM allows the detection of balanced and unbalanced chromosomal translocations, insertions, inversions, and copy number variants from only a few kbp in length up to a whole chromosome without cultivation, amplification, or sequencing. Recently, OGM has been shown to be equivalent to cytogenetics [12], and in another series of 27 adult AML cases, OGM was the technique of choice for newly defined karyotypes in 67% of that cohort [13]. Additionally, OGM has been shown to be a potential new tool in the diagnosis of other hematologic diseases such as MDS and pediatric ALL [13,14,15,16].

To the best of our knowledge, we here present for the first time OGM data on pediatric AML, which is known to exhibit a different molecular landscape in comparison to AML in adults [17,18]. Our aim was to test to what extent OGM may complement or even replace conventional cytogenetic tools at the initial diagnosis of pediatric AML. For this purpose, we analyzed material stored frozen from 24 children consecutively diagnosed with AML, bi-lineage leukemia, and mixed-phenotype acute leukemia (MPAL) and compared the results to karyotyping, including FISH analysis performed as part of the initial routine diagnostics.

## 2. Materials and Methods

### 2.1. Origin of the Samples Analyzed

Heparinized bone marrow aspirates (BMA, 5–10 mL) and peripheral blood (PB, 5–10 mL) were collected consecutively from children with suspected acute myeloid leukemia (AML) during the clinical routine when establishing the diagnosis. Samples were centrally evaluated, and the diagnosis was confirmed at the laboratory at University Hospital Essen, Germany, as part of the AML-BFM 2017 registry (DRKS number: DRKS00013030). All 24 samples (22 BMAs, 2 PB) included in this research project were taken from excess residual material collected during routine diagnostic procedures. The protocol was approved by the Institutional Ethics Board (University Hospital Essen, ethical vote number 17-7462-BO, 8 December 2017) and conducted in accordance with the Declaration of Helsinki. Written informed consent was obtained from all patients’ legal guardians and, if appropriate, by the patients receiving an age-adapted written explanation. An aliquot of 650 µL BMA and PB were taken from the samples and stored at −80 °C for later use. A total of 9.75 µL DNA stabilizer was added to the BMA aliquot before freezing.

### 2.2. Isolation of Ultra-High Molecular Weight DNA

UHMW gDNA extraction from frozen BMA was performed according to guidelines and with reagents provided by the manufacturer (Bionano Prep SP BMA DNA Isolation Protocol, Bionano Genomics #30298, Bionano Genomics, San Diego, CA, USA). A maximum of three samples was processed in parallel. Minor changes were applied as follows: instead of 1 mL BMA, only 650 µL were used as start material. Additionally, the cell number was determined using an automatic cell counter (Sysmex XP-300, SYSMEX SUISSE AG, Horgen, Switzerland). In short, after thawing, samples were pipetted on two separate BMA filters seated in tubes. Following centrifugation, the specific sample cell number was determined, and the volume was adjusted by adding cell buffer (Bionano Prep SP Blood and Cell DNA Isolation Kit) to achieve a concentration of 1.5 × 10^6^ cells diluted into a volume of 40 µL per sample.

Thereafter, proteinase K dissolved in lysis buffer, RNAse A, and LBB were added to lyse and digest the blood cells. The samples were rotated for 15 min on the HulaMixer (ThermoFisher Scientific, Waltham, MA, USA), followed by adding PMSF (Sigma-Aldrich, St. Louis, MO, USA) and 10 min of incubation at room temperature (RT). A Nanobind Disk and 340 µL isopropanol were added to each sample to precipitate the DNA and to ease DNA binding to the disk during a 15 min rotation on the HulaMixer. Thereafter, DNA was washed with buffers WB1 and WB2 and transferred into another tube. Elution buffer (EB) was added, and after 20 min incubation at RT, the specimens were gently rotated on the HulaMixer for 1 h. To further improve DNA rehydration and homogeneity, samples were stored for 1 to 2 days at RT. The two PB samples were processed according to the manufacturer’s guidelines (Bionano Prep SP Frozen Human Blood DNA Isolation Protocol v2, Bionano Genomics #30395), which does not include the first BMA filter step and starts with counting white blood cells.

### 2.3. Quantification of UHMW DNA

UHMW gDNA was quantified using the Qubit™ dsDNA BR Assay Kit (ThermoFisher Scientific, Waltham, MA, USA) and a Qubit 3.0 Fluorometer (ThermoFisher Scientific, Waltham, MA, USA). To check the gDNA’s homogeneity, concentration was determined from the top, the middle, and the bottom of the fluid in the tube. Extraction was rated as successful when the UHMW DNA concentration from the three collections was equal (coefficient of variation < 0.3) and above 36 ng/µL.

### 2.4. Labeling of UHMW gDNA and Chip Loading

Fluorescent dye labeling of the UHMW gDNA (specific labeling and whole DNA staining) was performed according to the manufacturer’s guidelines (Bionano Prep Direct Label and Stain (DLS) Protocol, Bionano Genomics, #30206). A total of 750 ng UHMW gDNA was labeled specifically at sequence CTTAAG with DL-green fluorophores using the DLE-1 enzyme. Proteinase K digestion (Qiagen, Hilden, Germany) was performed thereafter, followed by removing DLE-1 remnants with two different membranes on a microplate. As the next step, the whole gDNA was labeled by using DTT and DNA blue fluorescent stain (Bionano Genomics DNA stain reagent). The samples were rotated on the HulaMixer for 1 h, followed by incubation overnight at RT and protection from light. The labeled UHMW gDNA was quantified using the Qubit^TM^ dsDNA HS Assay kit (ThermoFisher Scientific, Waltham, MA, USA). To ensure homogeneity, specimens were collected at two different locations (e.g., top and bottom or left and right side) from each sample tube. A concentration in the range of 4 to 12 ng/µL was classified as acceptable with a coefficient of variation of the specimen < 0.25. Thereafter, the stained and labeled UHMW gDNA was loaded on a Saphyr G2.3 chip. By the Saphyr device, the DNA molecules were imaged with a maximum capacity of 1500 Gbp per sample.

### 2.5. Assemblies and Variant Calling

Structural variants were analyzed using the Bionano Access software (Tools version 1.6.1, Bionano Genomics, San Diego, CA, USA), making use of the rare variant analysis. After the software produced molecule files from the imaged DNA, the data were filtered to 1500 Gbp with a minimum length of 150 kbp. Rare variant analysis was then performed on each sample from these filtered molecular files. The data were then further filtered with recommended structural variant confidence filters, except for inversion, which we filtered as “all”. For the copy number variant (CNV) filter, a minimum size of 500 kbp was used. In four samples, the CNV confidence call was set to 0.1 after the whole CNV showed a visible deletion or duplication, which was not called by Bionano Access. The frequency of structural variants (SVs) that were detected in the Bionano control group was set to 0%. The self-molecule count (minimum number of molecules supporting the variant) was set to 5. As a reference, genome hg38 was used. Genuine translocation calls were distinguished from various SV artifacts, leading to rare translocation calls using manual inspection (Table S1). By manual inspection and comparison to data available from respective diagnostic reports, the clinical relevance for pediatric AML of the structural variation of each called SV was determined [6,7,9,10,19,20].

### 2.6. Data Comparison

The OGM results were compared to the known aberrations as detected by karyotyping, including FISH performed at the AML-BFM study reference laboratories in Hannover as described [19]. When comparing SVs and CNVs detected with OGM, we concentrated on aberrations clinically relevant for pediatric AML or relevant based on their frequency in reference genomes, excluding polymorphic regions present in the general population according to the database of genomic variants (DGV) [21]. The genes affected by deletions or duplications affecting more than one gene are listed in Table S2.

### 2.7. Validation of Newly Detected SVs by Long-Distance Touchdown PCR and Sequencing

Based on information from the Bionano analysis and the Snap Gene program (GSL Biotech LLC, San Diego, CA, USA), a map of the predicted deletions and translocations was generated. PrimerBlast was used to select suitable forward and reverse primers for each aberration (see Table S3) [22]. Long-distance touchdown PCRs [23] were performed using the GoTaq^®^Long PCR Master Mix Kit (Promega GmbH, Walldorf, Germany) following manufacturer recommendations. The expected PCR products with a length of 5 to 15 kbp were separated on a 0.8% agarose gel applying a 2.6 V/cm distance between the electrodes. Primer walking was used to amplify the breakpoint. The identified breakpoints were re-confirmed by a breakpoint-spanning PCR using the ALLin™ HS Red Taq Master Mix Kit (highQu GmbH, Kraichtal, Germany). Primers and annealing temperatures are listed in Table S3. The expected PCR products had a length of 600 to 1000 bp and were separated on a 2% agarose gel applying 8 V/cm distance between the electrodes. All PCR products were sequenced at a commercial lab (Microsynth Seqlab GmbH, Göttingen, Germany).

### 2.8. Applying Newly Detected SVs for Monitoring of Minimal Residual Disease (MRD)

Based on the new structural aberrations detected by OGM and confirmed by PCR, MRD monitoring was performed. In 2 of 3 cases exhibiting newly detected SVs, additional samples were collected at different time points during the antineoplastic treatment and were kept frozen in DMSO at −196 °C. For assessment of the lower limit of sensitivity, a logfold dilution series (10% to 0.001%) of the DNA sample from diagnosis into a healthy DNA sample was set up, and breakpoint-spanning PCR was performed as described above. For semiquantitative MRD monitoring during treatment, it was examined by visual comparison of band intensity at which dilution PCR products of translocations or deletions were still detectable at defined time points.

## 3. Results

Specimen collected at diagnosis from 24 patients with suspicion of pediatric AML were analyzed by OGM. Using routine methods such as morphology, karyotyping, and FISH, established diagnosis was primary AML in 20 cases, secondary AML in 2 cases, and each case of MPAL and bi-lineage AML (Table 1).

Following isolation of UHMW DNA, the quality standards regarding DNA length, cell count, and generated map counts were in the recommended range in 20 samples. Concerning the mean DNA length in the range of ≥200 kbp, two samples (case #14 and case #19) had a lower (132 and 141 kbp) mean length of the DNA molecules. However, as the mean length of the DNA molecules ≥ 150 kbp of the two samples was within the quality standard range, the rare variant analysis could be performed without problems. In the two other samples, the average label density (normal range 14 to 17) was either too high (case #10: 18.55) or too low (case #13: 13.17). Despite the deviation, the samples were further analyzed without problems. In total, >1.5 × 10^6^ molecules for each sample were aligned to the reference genome, resulting in an average effective coverage of the reference of 392×. The results presented were obtained using the rare variant pipeline with appropriate filter settings for confidence and size to prioritize SVs. Compared to classical cytogenetics, OGM detected a total of 32 additional SVs comprising in detail 13 deletions, 9 translocations, 7 duplications, 2 inversions, and 1 insertion that are known to be or might be clinically relevant were detected corresponding to 1.3 SVs per patient. The OGM findings in comparison to karyotyping and FISH of the individual patients are listed in Table 2.

**Figure 1:**
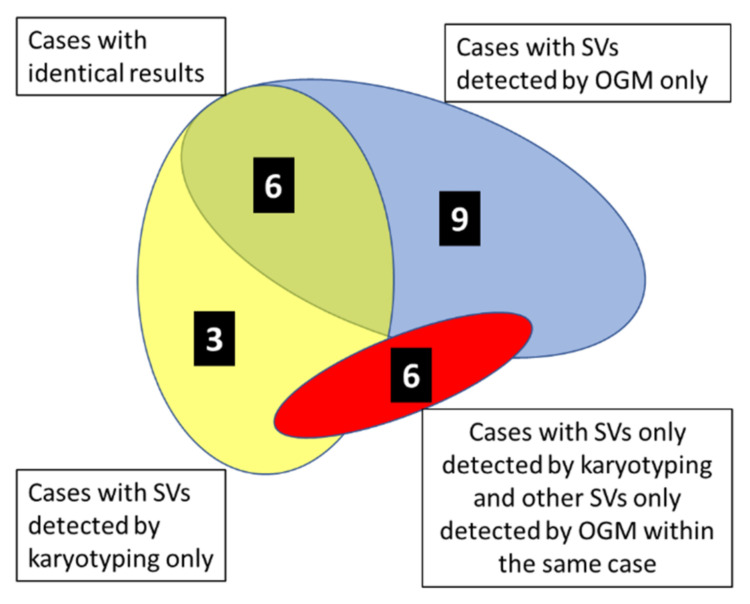
Diagram illustrating within the cohort of 24 cases of pediatric AML, 1 MPAL, and 1 bi-lineage leukemia the number of cases with identical results of karyotyping and OGM (N = 6, green overlapping area) and the number of cases exhibiting divergent results of karyotyping and OGM (as indicated). Colored areas are not according to scale.

As demonstrated by the diagram in Figure 1, results of karyotyping, including FISH when compared to OGM, were identical in 6 out of 24 (25%) cases analyzed (marked in green color in Table 2). In 3 out of 24 specimens (12.5%), SVs described by karyotyping were not detected by OGM (marked in yellow color in Table 2), and vice versa in 9 out of 24 specimens (37,5%), SVs were described by OGM but not by karyotyping (marked in blue color in Table 2). In 6 additional specimens (25%), SVs, as described by karyotyping, were not detected by OGM, but additionally, OGM detected SVs that were not described by karyotyping (marked in red color in Table 2).

### 3.1. Comparison of Karyotyping and FISH Results Versus OGM Results

#### 3.1.1. Cases with Identical Results

Findings of SVs unraveled by OGM and also by cytogenetic methods were identical in 6 of the 24 cases. In two cases (#18 and #23), both karyotyping and OGM revealed a normal karyotype. Three cases (#2, #7, #8) harbored a translocation t(8;21) associated with the well-known gene fusion *RUNX1*::*RUNX1T1*. In addition, case #2 exhibited the deletion del(9)(q21q31) on chromosome 9, and case #8 exhibited loss of the Y-chromosome. All these aberrations were confirmed by OGM. In case #17, monosomy 7 and trisomy 8 were described as cytogenetic findings, which were both confirmed by the CNV pipeline of OGM.

#### 3.1.2. Cases with SVs Detected by Karyotyping Only

In 3 patients (cases #1, #12, #20), SVs described by karyotyping were not detected by OGM. In case #1, OGM did not unravel monosomy 11, which was described by karyotyping in 3 out of 22 metaphases. However, trisomy 8, as described in 6 out of 22 metaphases by karyotyping, was also detected by OGM in this case. Due to that discrepancy, we performed an extended FISH diagnostic for monosomy 11, which did not unravel monosomy 11 and thus confirmed the OGM findings. In case #12, OGM did not unravel trisomy 8, which was described by karyotyping in 4 out of 21 metaphases. Here, we could not perform additional FISH analysis as there was no leftover material. In addition, karyotyping described a translocation t(9;11) causing the well-known fusion *KMT2A*::*MLLT3* in 16 out of 21 metaphases in case #12, which was also detected by OGM. In case #20, karyotyping and OGM showed a translocation t(11;19), resulting in the well-known fusion *KMT2A*::*MLLT1.* In addition, karyotyping described seven double-minute (dmin) chromosomes, which were classified as artifacts. Double-minute chromosomes were not present in the consecutive cytogenetic assessments performed during the course of treatment while the patient did not achieve remission (data not shown). OGM did not unravel double-minute chromosomes.

#### 3.1.3. Cases with SVs Detected by OGM Only

In 9 patients (#4, #9, #10, #13, #15. #16, #19, #21, #24), OGM detected additional aberrations that were not listed among the cytogenetic findings. In case #9, which was normal by karyotyping. OGM, however, detected a deletion of nearly 9 Mb at chromosome 1 between positions 44,994,758 and 53,861,278. The deletion has not been described before in pediatric AML. In case #24, also normal by karyotyping, OGM detected an insertion at chromosome 2 within the gene *TRIP*12 and additionally two duplications on chromosome 9 with one nearly 1.3 Mbp between positions 39,560,026 and 40,813,220 and one nearly 0.73 Mbp between positions 66,749,836 and 67,475,278. Case #4 harbored deletions at chromosomes 10 and 11, which were described by karyotyping as well as by OGM. However, only OGM showed a t(10;11) putative affecting genes *NRG3* and *FOXR1*. Additionally, an interstitial deletion del(19)(p13.11) of nearly 0.9 Mbp affecting the gene *MEF2B* was shown by OGM.

The cytogenetic findings in case #10 were a trisomy 8 and a trisomy 22 in 9 out of 20 metaphases and an inversion 16, causing the fusion *CBFB*::*MYH11* in 19 out of 20 metaphases. These aberrations were confirmed by OGM. In addition, OGM detected a deletion of nearly 22.3 Mbp on chromosome 7 spanning position 117,134,687 to 139,402,873. The low self-molecule count of 10 together with the CNV ratio of 1.8 for this deletion on chromosome 7 in the OGM analysis indicates a subclonal event.

In four cases (#13, #15, #16, #19) translocations were described by cytogenetics (case #13: t(10:11); case #16: t(15;17); case #15: t(17;19); case #19: t(6;9)) all of which were also detected by OGM. In addition, in case #13, OGM described a deletion on chromosome 11 (indicated as intrachromosomal translocation). In case #15, *MPO* (myeloperoxidase) and *PPP1R37* (protein phosphatase 1 regulatory subunit 37) were identified by OGM as potential fusion partners involved in translocation t(17;19). Additionally, OGM detected duplications on chromosome 2 and chromosome 8 and translocation t(2;12) involving the gene *ETV6* and the contig *AC064875.1*. In the remaining two cases (#16, #19), OGM detected extra duplications compared to karyotyping, namely a duplication of the whole q-arm of chromosome 13 in case #16 and duplication of cytoband p12 on chromosome 11 in case #19. In case #21, classical cytogenetics detected tetraploidy of chromosome 21 in 15 out of 20 metaphases and multiple deletions on chromosome 9. While the CNV pipeline of OGM confirmed the tetraploidy, the higher resolution of OGM showed are more complex and chromothriptic copy number pattern of chromosome 9.

#### 3.1.4. Cases with SVs Only Detected by Karyotyping and Other SVs Only Detected by OGM within the Same Case

In six cases (#3, #5, #6, #11, #14, #22), findings reported by karyotyping were not detected by OGM and vice versa within the same case. In case #3, karyotyping detected a deletion on the q-arm of chromosome 1 in 4 of 15 metaphases, which could not be confirmed by OGM. The detailed cytogenetic report classified this deletion as an event occurring during clonal evolution. Next to the translocation t(6;11) detected by both methods, OGM indicated 3 more translocations (t(2;6), t(4;8), and t(8;14)) as well as two deletions (del(2)(q36.3q37.2) and del(8)(p23.1q24.21)).

In case #5, karyotyping showed the translocation t(8;21)(q21;p13), which was not identified by OGM. There is an unbalanced translocation, and as the used OGM technique has no labels on the short arm of chromosome 21, it could only detect a gain of material on the long arm of chromosome 8. The translocation t(9;11) was confirmed by OGM. In addition, OGM detected translocation t(14;17), deletion del(14)(q24.3q32.33), and duplication dup(17)(q21.32q25.3) not seen by cytogenetics.

In case #6, karyotyping described t(7;13)(p11;p11) and t(1;8)(q21;p11). The latter was not identified by OGM due to missing labels in the centromeric regions of the chromosome. However, OGM detected gains of material on the long arms of chromosomes 1 and 8. Translocation t(7;13) was not detected by OGM. However, a translocation t(6;7) was described by OGM, which might represent the underlying cause for the derivative chromosome der(7) described by karyotyping. The deletion del(6)(q16q25) could not be confirmed by OGM. The translocation t(6;7) might explain the missing bands interpreted as del(6)(q16q25) in the cytogenetic report. Trisomy 10 was shown by karyotyping and OGM.

In case #11, karyotyping discovered a trisomy 21 in 2 metaphases, a trisomy 6 and 20 in 5 metaphases, and tetraploidy in one metaphase, which were classified in the cytogenetic report as probable artifacts. To clarify this, we performed an extended FISH diagnostic and confirmed the results. However, these aberrations were not detected by OGM. Instead, OGM showed an inversion on chromosome 16, representing the putative gene fusion *GLIS2*::*CBFA2T3,* which was confirmed by RNA fusion analysis (for details, see Section 3.3). In case #14, karyotyping detected an additional chromosomal material on chromosome 11. This was not confirmed by OGM; however, in the OGM analysis, an unbalanced translocation t(11;17) and an inversion on chromosome 3 were described. The tetraploidy of chromosome 6 was found in both karyotyping and OGM analysis. In case #22, karyotyping and OGM detected the translocation t(17;19). While karyotyping detected an additional part on chromosome 13, OGM found a deletion on chromosome 13. Furthermore, karyotyping showed a der(4)(del(4)(p11p15)del(4)(q11q24). OGM, however, detected deletions del(4)(p12) and del(4)(q12q13.1). Additionally, two translocations (t(4;7), t(8;12)) and 4 deletions (del(1), del(15), del(16), del(22)) were found by OGM only.

### 3.2. Successful Validation of 3 Novel OGM Findings by Breakpoint-Spanning PCR

As OGM is a relatively new method, we validated the presence of three selected SVs (one deletion and two translocations). Using the information of the last labels adjacent to the deletions or fusion breakpoint, we designed primers and performed long-distance PCRs. The generated products ranging in size from 5 to 15 kbp were subsequently sequenced, and primer walking was applied until the deletion or breakpoint regions were covered. The sequence information was used to design deletion and breakpoint-spanning PCRs to validate the presence of the SVs.

**Figure 2:**
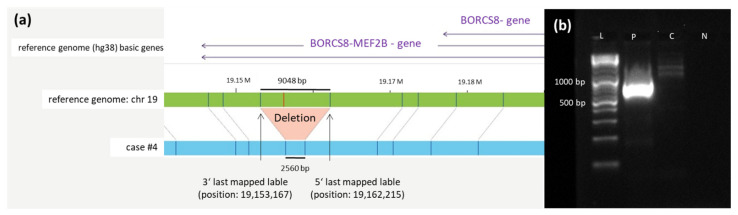
Deletion at chromosome 19. (**a**) Schematic representation of the newly identified deletion on chromosome 19 in case #4 detected by OGM. The first bar represents the reference genome (hg38) basic genes located at the depicted position (*BORCS8-MEF2B*). The second bar (green) shows the reference genome map of chromosome 19. The last bar (blue) represents the map of case #4. Vertical blue lines represent mapped labels, whereas red vertical lines represent missing labels in case #4 indicating the deletion. (**b**) Validation of the chromosome 19 deletion in case #4 via PCR. Deletion-spanning PCR was performed on genomic DNA of case #4 using forward and reverse primers (see sequences given in Table S3), generating a product with the expected length of 792 bp. A healthy control-DNA and the negative control were both negative in the PCR. Abbreviations: C = control-DNA; chr = chromosome; hg38 = human genome project 28; L = length marker; N = negative control (H_2_O); P = sample from case #4 collected at diagnosis; RNF = ring finger protein.

The deletion at chromosome 19 described in case #4 is 6488 bp long (last mapped label start at 19,153,167, end at 19,162,215) and affects the gene *MEF2B*, as shown in Figure 2.

**Figure 3:**
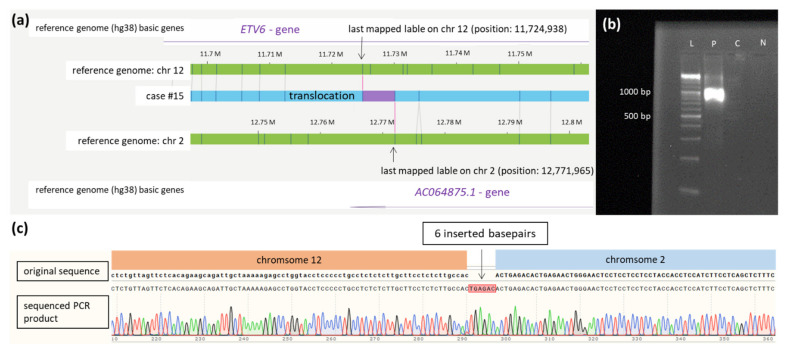
Translocation t(2;12). (**a**) Schematic representation of the newly identified translocation t(2,12) in case #15 detected by OGM. The first bar represents the reference genome (hg38) basic genes of chromosome 12 located at the depicted position (*ETV6*). The second and fourth bars (green) show the reference genome map of chromosome 2 and chromosome 12. The third bar (blue) represents the map of case #15. The fifth bar represents the reference genome (hg38) basic genes of chromosome 2 located at the depicted position (*AC064875.1*). Vertical blue lines represent mapped labels. (**b**) Validation of the translocation t(2;12) in case #15 via PCR showing a product of 1138 bp spanning translocation t(2,12) (ranging from 12,767,669 on chromosome 2 to 11,725,810 on chromosome 12). The healthy control-DNA and the negative control were both negative in the PCR. (**c**) Sequenced PCR product spanning the breakpoint of translocation t(2;12) in case #15 with insertion of 6 base pairs. In the first row, the original sequence of chromosome 12 followed by chromosome 2 is shown (modified from the figure as shown in the SnapGene program^TM^). The second row depicts the electropherogram and sequence of PCR product of case #15 aligned to the original sequences spanning the breakpoint of the translocation. Insertion of 6 base pairs (marked red) at the breakpoint is shown in the middle of the second row and probably results from DNA repair mechanisms at the breakpoint. Abbreviations: C = control-DNA; chr = chromosome; hg38 = human genome project 38; ETV = ETS variant transcription factor 6; L = length marker; N = negative control (H_2_O); P = sample from case #9 collected at diagnosis.

Translocation t(2;12) affecting the gene *ETV6* at chromosome 12 and the contig *AC064875.1* on chromosome 2 were confirmed by breakpoint-spanning PCR. The precise breakpoints could be determined to positions 12,767,669 on chromosome 12 and 11,725,810 on chromosome 2, respectively, including insertion of 6 bp (see Figure 3).

**Figure 4:**
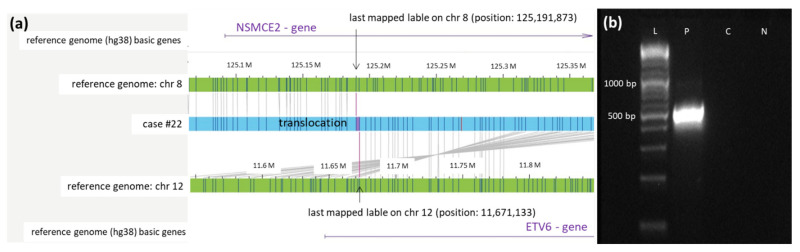
Validation of the newly identified translocation t(8;12) in case #22 detected by OGM. (**a**) Schematic representation of the newly identified translocation t(8,12) in case #22 detected by OGM. The first bar represents the reference genome (hg38) basic genes of chromosome 8 located at the depicted position (*NSMCE2*). The second and fourth bars (green) show the reference genome map of chromosome 8 and chromosome 12. The third bar (blue) represents the map of case #22. The fifth bar represents the reference genome (hg38) basic genes of chromosome 12 located at the depicted position (*ETV6*). Vertical blue lines represent mapped labels. (**b**) Validation of the translocation t(8;12) in case #22 via PCR. PCR was performed using forward and reverse primers (see Table S1), generating a product of 513 bp spanning translocation t(8,12) (ranging from position 125,191,873 on chromosome 8 to position 11,671,133 on chromosome 12). The healthy control-DNA and the negative control were both negative in the PCR. Abbreviations: C = control-DNA; chr = chromosome; hg38 = human genome project 38; ETV = ETS variant transcription factor 6; NSMCE2 = SMC5-SMC6 complex SUMO ligase; L = length marker; N = negative control (H2O); P = sample from case #9 collected at diagnosis.

In case #22, a translocation t(8;12) was described by OGM. The translocation affects the genes *NSMCE2* on chromosome 8 and *ETV6* on chromosome 12. Sequencing of the PCR product located the precise breakpoints at 125,191,873 on chromosome 8 and 11,672,133 on chromosome 12 (Figure 4).

### 3.3. Stratification of Pediatric AML by Cytogenetics Versus OGM

Within the total cohort of 24 patients, the two cases of secondary AML (#3, #20) and each the case of MPAL (#24) and bi-lineage leukemia (#15) were categorized upfront as high-risk leukemias according to AML treatment protocols without requirements for additional assessment of cytogenetic markers. The remaining 20 patients with de novo AML were stratified for the risk of treatment failure or relapse based on cytogenetic findings according to the AML-BFM criteria [19]. As can be taken from Table 1, 5 patients each were assigned to the standard and intermediate-risk group, and 10 patients to the high-risk group. Performing the same stratification using the results generated by OGM, no change in the grouping for risk stratification resulted in 19 of the 20 patients. Importantly, patient #11, carrying the high-risk marker inv(16) with a fusion of *GLIS2*::*CBFA2TA,* had to be regrouped from intermediate risk into the high-risk group. The presence of the fusion was confirmed by routinely performed RNA sequencing using the Illumina TruSight RNA Fusion Panel.

### 3.4. MRD Monitoring Based on Validated Novel OGM Findings

**Figure 5:**
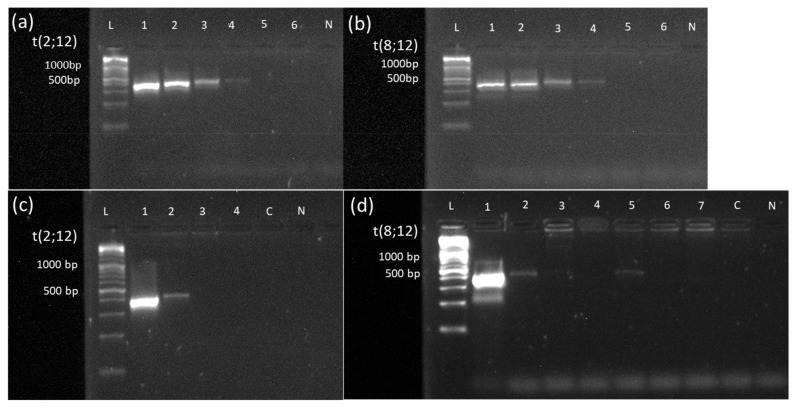
Sensitivity assessment of PCR-based MRD monitoring via a dilution series in cases #15 and #22 and Monitoring of MRD based on translocation t(2;12) and t(8;12) newly detected by OGM. A 10-logfold dilution series (100% to 0.001%) of the DNA sample from diagnosis into a healthy DNA sample was performed in cases #15 (**a**) and #22 (**b**). Specimen from the dilution series were subjected to breakpoint-spanning PCR with forward and reverse primers, as indicated in Table S1. In both cases, the PCR becomes negative at the 0.01% dilution step (Lane 5), indicating a sensitivity limit of detecting one blast within 1000 healthy cells (Lane 4). (**c**) MRD monitoring via breakpoint-spanning PCR in case #15. PCR was performed using forward and reverse primers (see Table S3), generating a product of 389 bp spanning the breakpoint of translocation t(2,12). Samples were collected at initial diagnosis (1), on day 28 after the start of treatment (2), after AI treatment (3), and after hAM treatment (4). PCR was positive at initial diagnosis and on day 28 after the start of treatment. Visual comparison of the band to the dilution series unraveled MRD in the range of 1%. At all other time points, as well as healthy control-DNA and negative control were negative in PCR. (**d**) MRD monitoring via breakpoint-spanning PCR in case #22. PCR was performed using forward and reverse primers (see Table S3), generating a product of 380 bp spanning the breakpoint of translocation t(8,12). Samples were collected at initial diagnosis (1), on day 28 after the start of treatment (2), after HAM treatment (3), after AI treatment (4), after hAM treatment (5), on day 60 (6), and on day 100 after SCT (7). PCR was positive at initial diagnosis, on day 28 after the start of treatment, and after hAM treatment. Healthy control-DNA and negative control were both negative. Abbreviations: 1 = 100% patient DNA; 2 = 10% patient DNA; 3 = 1% patient DNA; 4 = 0.1% patient DNA; 5 = 0.01% patient DNA; 6 = 0.001% patient DNA; C = control-DNA; chr = chromosome; L = length marker; N = negative control (H_2_O), t = translocation.

In cases #15 and #22, frozen DNA samples collected during the course of antileukemic treatment were available. The translocation breakpoint of t(2;12) was used for MRD monitoring in case #15, whereby primers amplifying a PCR product with a length of 389 bp were used (Table S3). Samples collected on day 28 after treatment, after AI treatment, and after hAM were available. The PCR was positive at initial diagnosis and on day 28 after the start of treatment, while PCR was negative on all later time points (Figure 5c). Visual comparison of the intensities of the band on day 28 after the start of treatment to the dilution series (Figure 5a) unraveled MRD in the range of 1%. In morphologic analysis, blasts were only detected at the initial diagnosis. For translocation t(8;12) in case #22, the breakpoint-spanning PCR for MRD monitoring generated a product of 380 bp (Table S3). Samples analyzed from initial diagnosis, day 28 of initial treatment, and after hAM were positive in PCR (Figure 5d). However, a visual comparison of the intensities of bands on day 28 of initial treatment and after hAM to the dilution series (Figure 5b) unraveled MRD in the range of 0.1% to 0.01%. At all other time points (after HAM treatment, after AI, on day 60, and on day 100 after SCT), MRD monitoring stayed PCR negative. In the morphologic analysis, 90% of blasts were detected at the initial diagnosis, 23% after 28 days of initial treatment, and 0% after hAM, respectively.

Thus, MRD monitoring with markers identified by OGM showed the potential to allow MRD monitoring in selected patients.

## 4. Discussion

AML in adult and pediatric patients is characterized by genetic heterogeneity of the molecular landscape and clonal evolution of the leukemic stem cell [24,25]. When diagnosed, microscopic morphology of AML blasts, karyotyping, complemented by FISH technique, immunophenotyping, and RT-PCR for currently known specific genetic alterations [26] all form the cornerstones for assessment of distinct prognostic outcomes in a given patient, and consequently, the selection of an appropriate treatment protocol. The specific type of a single genetic aberration, as well as the cumulative number of aberrations both, have an impact on the stratification into standard-, intermediate-, or high-risk groups [6]. Thus, for an optimal diagnosis of AML and also for a better understanding of treatment response, it is of utmost importance to detect chromosomal structural variants as precisely as possible.

Classical cytogenetic methods have achieved great things on the long road to improving the prognosis of childhood AML [1]. However, karyotyping is not free from problems and limitations, among which are a relatively high need for time, cost-intensiveness, necessity of experienced and intensively trained staff, and the availability of living cells.

OGM was recently introduced as a potential new tool for the diagnosis of malignant hematology in adult AML, ALL, MDS, and pediatric ALL [13,14,15,16]. In the study presented here, we asked the question of whether and to what extent OGM might overcome the disadvantages of karyotyping in the routine workflow of an AML pediatric reference laboratory. Key questions asked were the practicability of OGM and whether this new technique might be able to replace karyotyping and FISH analysis. We performed OGM analysis on specimens stored frozen from 24 children consecutively diagnosed with AML, bi-lineage leukemia, and MPAL and retrospectively compared the OGM findings to the individual reports based on the routine workflow of karyotyping and FISH technique at diagnosis. The patient cohort examined represents by number and AML subtypes the typical spectrum of pediatric AML as diagnosed during a 3-month period in Germany [1].

### 4.1. Practicability

The result of karyotyping depends on the number of cells that can be used for cultivation. One advantage of OGM is the ability to obtain DNA directly from patient samples without the need for cell cultivation or amplification steps. The handling of UHMW DNA requires experience, especially in pipetting. In addition to using wide-bore pipette tips, it is essential to aspirate the liquid very slowly and carefully in order to minimize the shear forces acting on the DNA. However, these and all other steps can be learned and mastered within a few weeks, even by untrained staff. At two steps in the workflow of the protocol, quality of the UHMW DNA is assured with respect to concentration, and in the last step (chip loading with subsequent DNA scanning), a molecule quality report (MQR) is generated. This includes molecule quality values such as N50 of DNA molecules with a length of ≥150 kbp, label density (average number of labels per 100 kbp for molecules sized ≥ 150 kbp), effective coverage (total amount of aligned DNA divided by the size of the reference genome times the map rate) and map rate (percentage of molecules that are ≥150 kbp and mapped to the reference). These molecule quality standards were not met by four samples (17%) [27]. Nevertheless, the samples could be analyzed without problems, so no efforts were made to evaluate the limit of the quality standards recommended by Bionano Genomics.

### 4.2. Higher Resolution

Methods used to detect genetic aberrations vary in their resolutions. Karyotyping has a resolution of 4 to 5 Mbp, which excludes a more precise assignment of affected genes. FISH diagnostics has the advantage of a much higher resolution in the range of 100 kbp. However, it is not a genome-wide approach, and its application is limited to specific, already known aberrations. OGM clearly exceeds the limitations of both methods with a genome-wide resolution down to 500 bp [28]. Thus, the high resolution of OGM is an important extension of the methodological spectrum, which resulted in our cohort in the detection of new SVs not previously identified with the established methods in 15 of 24 patients (62.5%). This corresponded to approximately 1.3 additional SVs per patient. This number of newly detected aberrations is consistent with the experience of other authors. Neveling et al. [16] found in a cohort of 52 cases with varying hematological malignancies a non-specified wide range of additional SVs. Almost identically to our findings, Gerding et al. [13] identified 1.2 SVs per case (33 new SVs in 27 cases) in adults with AML/MDS, and Lestringant et al. [14] found 16 anomalies in 10 cases of adults with ALL (1.6 SVs per case), which were not found by any other technique.

As already described by Gerding et al. [13], another possibility of OGM, based on the high resolution and genome-wide analysis, is the possibility of detecting complex karyotypes, marker chromosomes, and chromothriptic events. For example, in case #21, the higher resolution of OGM identified chromothripsis as the underlying cause of the chromosomal finding of multiple deletions on chromosome 9.

In cases #14 and #22, the OGM refined the analysis of classical cytogenetics, which only described the SVs as additional chromosomal material by assigning the SVs to defined chromosomes in the karyotyping. In case #14 the add (11) corresponded to a translocation t(11,17) and in case #22 an add(13) corresponded to a deletion del(13)(q14.2q14.3).

### 4.3. Subclones

In pediatric AML, subclones are often generated by the accumulation of different genetic aberrations [19,24,25]. Therefore, a disadvantage of karyotyping is the initial cultivation of the cells, which can lead to some cells gaining a proliferation advantage. After arresting proliferation in metaphase, only a relatively small number of usually 20 metaphase plates are examined for existing chromosomal aberrations. Thus, there is a risk of missing subclones or false quantification of the relative proportion of different populations of subclones by the biased selection of metaphases [29,30]. This could result in incorrect risk stratification of patients. OGM examines the DNA of all cells; however, the capability to detect SVs in the subclones is strongly influenced by the cut-off values of the rare variant pipeline (usually recommended) and CNV pipeline (usually confidence: 0.99) filter settings.

In our cohort, OGM failed to describe SVs in 9 of 24 cases (37.5%) detected by conventional methods. As shown in cases #1, #11, and #12, OGM is worse at detecting aneuploids, especially in subclones. However, these SVs have only been found in a few metaphases of each case (3/22, 5/26, and 4/22 for cases #1, #11, and #12, respectively). In case 1, no monosomy 11 was detected in an extended FISH analysis, suggesting that it was indeed a cultural artifact in the karyotyping. In case 11, however, the additional FISH diagnosis confirmed trisomy 6, 20, and 21 in 19, 16, and 21, respectively, cells out of 100 cells analyzed. OGM could not detect this subclone. For case 12, no extended FISH analysis could be performed due to a lack of material. What the exact limit of OGM for the detection of subclonal aneuploidy is cannot be deduced from our non-systematic study with only two discrepantly described and thus very small numbers of cases. In case #1, karyotyping described trisomy 8 in 6 of 22 metaphases (27%), which could be detected by OGM only by visual inspection of the CNV pipeline (see Figure S1). Lowering the confidence filter of the CNV to 0.1 led to the detection of this aberration by OGM. Similar cases were also observed by Gerding et al. [13] and Lestringant et al. [14]. However, the changes visible by visual inspection, as in case #1, are not always called out as CNV. Here, Bionano Access Server needs further improvement in the filter settings to ensure that these CNVs are not only detected by visual inspection.

### 4.4. Blast Count

The manufacturer Bionano claims to detect aberrations in the CNV pipeline up to a VAF of 10% [28], but it has to be stressed that for achieving this sensitivity, a blast count of 100% is required. However, a considerable proportion of AML patients may have relatively lower blast counts [1]. In our cohort, 4 out of 24 patients (15%) (#2, #6, #14, #17) had a blast count > 20% but <30% in PB or BMA. Since straight aneuploidy can only be identified in the CNV pipeline up to a VAF of 10% with OGM when the blast count is 100%, this is an important factor. The rare variant pipeline can detect SVs up to a VAF of 1% with OGM. In our cohort, one patient (#2) had a blast count of only 22% and another case (#17) of only 26%, both resulting in SVs findings consistent with karyotyping. Thus, it can be assumed that OGM using the rare variant pipeline can provide reliable results even at blast counts < 30%. However, special attention should be paid to CNV filter settings at low blast counts, and visual inspection of the CNV pipeline is essential.

### 4.5. Cultural Artifacts

The alterations detected during karyotyping in a small proportion of metaphases were classified as cultural artifacts in three patients (#1, #12, #3). The cytogenetically described monosomy 11 in case #1, which was confirmed to be an artifact by additional FISH analysis, and also the deletion on chromosome 1 found in case #3 are both not typical aberrations in pediatric AML [1,6]. None of these aberrations was found by OGM. In case #20, double-minute chromosomes, which were described as possible artifacts in the cytogenetic findings, were reliably identified as such by OGM. Due to the higher resolution of OGM, it is possible to identify potential cultural artifacts of karyotyping. As shown in Gerding et al. [13], OGM can even define unclassified marker chromosomes.

### 4.6. OGM Has No Markers on Defined Chromosome Segments

In cases #5 and #6, two translocations are defined by cytogenetics only, which were not detected by OGM due to limitations of the used DNA fluorescence labeling. The labeling applied by the manufacturer for the OGM methodology exploits the short base motif CTTAAG, which, however, is not present on the p-arm of acrocentric chromosomes (chromosomes 13, 14, 15, 21, and 22) [15,31]. Thus, translocations or CNVs of the p arms present on these chromosomes cannot be detected by the currently used OGM methodology. Furthermore, maps cannot be generated in highly repetitive regions. For example, this is shown in case #6 by the missed detection of translocation t(1;8)(q21;p11) in the centromeric region of chromosome 8.

### 4.7. Newly Discovered SVs

Precise localization of the position of the last known labels upstream and downstream of the SVs described in the Bionano Access Server provided the opportunity to validate newly found aberrations using primer walking and breakpoint-spanning PCR. Three of these newly described SVs could be precisely localized using long-range PCR and Sanger sequencing. SVs present in the *TRIP12* and *MEF2B* genes could be precisely defined using OGM. In mantel cell lymphoma, *MEF2B* expression has been shown to correlate with pathological subtypes, structural subtypes, and the expression of *SOX11*, but not with proliferation and prognosis [32]. The role of *MEF2B* deletions in pediatric AML needs to be clarified by further studies.

The translocations newly described in cases #15 and #22 by OGM involve the gene *ETV6* on chromosome 12. *ETV6* is a known regulatory factor of hematopoiesis, and *ETV6* rearrangements are described in many hematological malignancies [33]. The authors de Braekeleer et al. [33] stated that there are five potential mechanisms how *ETV6* can contribute to leukemogenesis: constitutive activation of the kinase activity of the partner protein, modification of the original functions of a transcription factor, loss of function of the fusion gene, affecting *ETV6* and the partner gene, activation of a proto-oncogene in the vicinity of chromosomal translocation and dominant negative effect of the fusion protein over transcriptional repression mediated by wild-type *ETV6*.

In case #15, a potential fusion of *ETV6* with the contig *AC064875.1* on chromosome 2 was detected by OGM. Although not comprising any genes, contig *AC064875.1* contains some lncRNAs and putative genes, which might lead to an alteration of *ETV6* expression or regulation [34]. In case #22, translocation leads to a potential fusion of *ETV6* with *NSMCE2*. *NSMCE2* is an SMC5-SMC6 complex SUMO ligase that has been studied in mice in the context of cancer suppression and aging [35]. Further functional analyses are planned to shed light on the involvement of these new fusion partners with *ETV6* in AML leukemogenesis.

### 4.8. Newly detected SVs used as MRD marker

Due to the very high resolution of OGM, it is possible to detect new SVs, with the potential to be used as MRD markers if required. This holds especially true for cases where none of the previously known markers for PCR-based MRD monitoring could be identified (approximately 30% of initial pediatric AMLs in Germany, data not shown).

As shown here in cases #15 and #22, OGM allows identifying a suitable MRD marker and using it for MRD monitoring with significantly higher sensitivity compared to morphology [36]. While in case #15 no blasts were found morphologically 28 days after the start of therapy, residual disease of about 1% could be detected by PCR analysis with a sensitivity threshold of 0.1% (see Figure 5). In case #22, a blast count of 23% was found in the morphology on day 28 after the start of therapy, which could be confirmed by PCR analysis, although the intensity of the band indicated a lower percentage of blasts. At all subsequent time points, no more blasts were detected by morphology in this case. In contrast, MRD monitoring by PCR showed residual disease of about 0.1% after hAM treatment displaying the potency of the method to detect MRD during treatment and to adjust therapy if needed (see Figure 5).

### 4.9. Changes in Risk Classification

Cases with secondary AML, MPAL, and bi-lineage leukemia are categorized independently from the presence of SVs upfront as high-risk leukemias solely based on the case history or the immunophenotype, respectively. Therefore, the analysis of any resulting change in risk classification when comparing classical karyotyping with OGM was performed only in the remaining 20 cases with de novo AML. In 19 of the 20 patients from this cohort, OGM did not change the risk stratification according to the AML criteria [1,19]. Cytogenetics detected SVs in 9 of 24 cases, which could not be confirmed by OGM (see Table 2). In case #1, less than three structural aberrations were present in total; thus, the patient was classified in the intermediate-risk group according to cytogenetics. With OGM, only one SV was described in these cases instead of two SVs. Nevertheless, this non-detection of one SV did not change the risk stratification. The patient would still have been correctly stratified by OGM. In case #20, the patient was assigned to the high-risk group by the presence of secondary AML after Ewing sarcoma alone, so failure to detect double-minute chromosomes by OGM did not affect assignment. In 6 cases (#3, #5, #6, #12, #14, #22), patients were assigned to the high-risk group by the number of SVs or by a specific high-risk SV. By OGM, other mutations were found that did not change the number > 3 SV for high-risk stratification. On the other hand, in all these cases, high-risk mutations were also detected by OGM, which means that failure to detect other SVs did not affect stratification. In case #11, on the other hand, the detection of inv(16) by OGM only resulting in gene fusion *GLIS2*::*CBFA2T3* required a regrouping into the high-risk group.

Thus, in the majority of cases, OGM was equally suitable to perform proper risk stratification concerning genetic aberrations compared to classical cytogenetics and, in rare cases, can lead to an optimization of therapy from the time of diagnosis. Thus, performing retrospective studies with OGM on preserved material from patients with poor response to therapy or AML relapse in the low-risk group could clarify whether intensification of therapy would have been appropriate already at the time of diagnosis.

## 5. Conclusions

Our study shows that OGM has much potential to address the limitations of cytogenetics, but some shortcomings of OGM still exist. Although discrepant results between classical cytogenetics and OGM were observed in a significant proportion of cases, these discrepancies had almost no effect on patients’ care in terms of risk stratification. Nevertheless, future studies with larger cohorts will further increase the trust and reliability of OGM and will also lead to improved evaluations of the complex data requiring, e.g., better filter settings for CNV calls of subclones. In addition, studies focusing on combining OGM results with sequencing data, e.g., panel-sequencing, have the possibility to improve risk stratification, leading to an enhanced patient treatment [37]. However, nowadays, the higher resolution and whole-genome approach of OGM allows the identification of many new aberrations that were previously undetected and thus results in new insights into the leukemogenesis of pediatric AML. In addition, OGM offers the possibility to identify new aberrations that can serve as patient-specific MRD markers in cases where no previously known MRD markers are present. This evidently will leverage a great benefit for guiding the treatment of these patients.

## Figures and Tables

**Table 1:** Patient’s characteristics, proportion of leukemic blasts among nucleated cells in the sample analyzed, subtype of leukemia according to the FAB classification, and resulting risk group categorized to the AML-BFM criteria.

Pat.#	Sex	Age (Years)	Blast Count/Source	FAB Type	Risk Group *
1	m	17	48% BM	M2 Auer positive	intermediate
2	m	10	22% BM	M2	standard
3	f	3	44% BM	sec. AML	high
4	m	16	97% BM	M5	high
5	f	1	87% BM	M4	intermediate
6	m	1	26% BM	M7	high
7	m	16	65% BM	M2 Auer positive	standard
8	m	13	38% BM	M2 Auer positive	standard
9	m	17	88% BM	M2	intermediate
10	f	14	71% BM	M4 eo	standard
11	f	2	90% BM	M7	high
12	m	3	43% BM	M5	intermediate
13	f	5	95% BM	M5	high
14	m	1	23% BM	M7	high
15	m	5	82% BM	Bi-lineage leukemia	not applicable **
16	m	16	88% BM	M3 Auer positive	not applicable **
17	m	7	26% BM	M2	high
18	m	11	48% BM	M2	standard
19	f	13	43% PB	M2	high
20	f	5	73% PB	sec. AML after Ewing’s sarcoma	high
21	f	5	90% BM	M1 Auer positive	high
22	m	5	90% BM	M1 Auer positive	high
23	m	10	48% BM	M1	intermediate
24	f	13	91% BM	MPAL	not applicable **

* according to AML-BFM study protocol 2012 (EudraCT number: 2013-000018-39). ** therapy related to AML-BFM study protocol 2012. Abbreviations: AML = acute myeloid leukemia; BM = bone marrow; BFM = Berlin, Frankfurt, Münster Study Group; eo = eosinophils; f = female; FAB = French-American-British classification; m = male; MPAL = mixed-phenotype acute leukemia as defined by two (or more) subclones expressing different immunophenotypes. In contrast, bi-lineage leukemia denotes the simultaneous expression of, e.g., lymphoid and myeloid markers by one clone; PB = peripheral blood; Pat.# = patient number.

**Table 2:** Comparison of karyotyping and FISH versus OGM.

Pat. #	Karyotyping	FISH	OGM (hg38) ^1^	OGM Predicted Karyotype
2	46,XY,t(8;21)(q22;q22),del(9)(q21q31) [8]/46,XY[7]	nuc ish 8q22(ETOx3),21q22(AML1x3)(ETO con AML1x2)[65/100]	ogm[GRCh38]46,XY,t(8;21)(q22.1;q22.12)(92059784;34850575),9q21.11q31.1(67717842_102668165)x1	46,XY,t(8;21)(q22.1;q22.12),del(9)(q21.11q31.1)
7	46,XY,t(8;21)(q22;q22)[13]/46,XY[2]	nuc ish 8q22(ETOx3),21q22(AML1x3)(ETO con AML1x2)[83/100]	ogm[GRCh38]46,XY,t(8;21)(q21.3;q22.12)(92067075;34843977)	46,XY,t(8;21)(q21.3;q22.12)
8	45,X,−Y,t(8;21)(q22;q22)[14]/46,XY[1]	nuc ish cen7(CEP7x2),7q31(D7S486x2[100/100],cen8(CEP8x2)[99/100),8q22(ETOx3),21q22(AML1x3)(ETO con AML1x2)[95/100]	ogm[GRCh38]45,X,t(8;21)(q21.3;q22.12)(92059784;34855785),Yp11.32q12(11554_57212132)x1~2	45,X,-Y,t(8;21)(q21.3;q22.12)
17	45,XY,−7[14]/46,idem,+8[6]	nuc ish 8q22(RUNX1T1x3),21q22(RUNX1x2)[12/100]	ogm[GRCh38]45,XY,7p22.3q36.3(10487_159334984)x1,8p23.3q24.3(61806_145076125)x2~3	46,XY,-7,+8 ^2^
18	46,XY[25]	negative	ogm[GRCh38]46,XY	46,XY
23	46,XY[25]	negative	ogm[GRCh38]46,XY	46,XY
1	47,+8[6]/45,XY,−**11**[3]/46,XY[13]	nuc ish 8q22(RUNX1T1x3),21q22(RUNX1x2)[13/100] nuc ish 11q23(MLLx2)[100/100] 3	ogm[GRCh38]47,XY, 8p23.3q24.3(208898_145076125)x2~3	47,XY,+8
12	46,XY,t(9;11)(p21;q23)[16]/ 47,idem,**+8**[4]/46,XY[1]	nuc ish 11q23(MLLx2)(5′MLL sep 3′MLLx1)[15/100]	ogm[GRCh38]46,XY,t(9;11)(p21.3;q23.3)(118493942;20375121)	46,XY,t(9;11)(p21.3;q23.3)
20	46,XX,t(11;19)(q23;p13)[8]/ 47,idem,**+dmin**[7]	nuc ish 11q23(MLLx2)(5′MLL sep 3′MLLx1)[77/100]	ogm[GRCh38]46,XX,t(11;19)(q23.3;p13.3)(118479068;6205232)	46,XX,t(11;19)(q23.3;p13.3)
4	46,XY,del(10)(q21q22),del(11)(q23)[12]/46,XY[3]	nuc ish (MLLx1)(5′MLL sep 3′MLLx1)[95/100]	ogm[GRCh38]46,XY,**10p12.31(20591034_21642851)**x**1**,10q21.1(56175397_57570855)x1,10q21.1q21.2(58331941_62944232)x1,10q22.2(73474864_75389039)x1,10q23.1(82757631_85834864)x1,**t(10;11)(q21.1;q23.2)(58331941;113938345),19p13.11(19153167_19162215)**x**1**	46,XY,der(10;11)t(10;11)(q21.1;q23.2),**del(19)(p13.11)**
9	46,XY[25]	negativ	ogm[GRCh38]46,XY,**1p34.1p32.3(44994758_53861278)**x**1**	46,XY,**del(1)(p34.1p32.3)**
10	46,XX,inv(16)(p13q22)[10]/ 48,idem,+8,+22[9]/46,XX[1]	nuc ish 8q22(RUNX1T1x3),21q22(RUNX1x2)[36/100],16q22(CBFBx2)(5′CBFB sep 3′CBFBx1)[64/100]	ogm[GRCh38]48,XX,**7q31.2q34(117134687_139402873)**x**1,**8p23.3q24.3(61805_145076125)x2~3,16p13.11(15709259_16475295)x1,16p13.11q22.1(15706265_67104157)inv,22p13q13.33(10514803_50805587)x2~3	48,XX,+8,+22, **del(7)(q31.2q34) ^4^**,del(16)(p13.11),inv(16)(p13.11q22.1)
13	46,XX,t(10;11)(p12;q23)[13]/46,XX[7]	nuc ish 11q23(MLLx2)(5′MLL sep 3′MLLx1)[96/100]	ogm[GRCh38]46,XX,t(10;11)(p12.31;q23.3)(21653601;108120278),**11q22.3q23.3(108113047_118493942)x1**	46,XX,t(10;11)(p12.31;q23.3),**del(11)(q22.3q23.3)**
15	46,XY,?t(17;19)(q22;q13)[20]	nuc ish 12p13(ETV6x3),21q22(RUNX1x2)[85/100],16q22(CBFBx2)[99/100],17q21.1(RARAx2)[98/100],19p13(E2Ax2)[98/100]	ogm[GRCh38]46,XY,**2p11.2(87120538_87736106)x2~3,t(2;12)(p24.3;p13.2)(12771965;11724938),8q23.1(108722194_109473788)x2~3**,t(17;19)(q22;q13.32)(58275730;45095385)	46,XY,**dup(2)(p11.2),t(2;12)(p24.3;p13.2),dup(8)(q23.1),**t(17;19)(q22;q13.32)
16	46, XY,t(15;17)(q24;q21),inc[15]	nuc ish 17q21.1(5′RARAx3,3′RARAx2)(5′RARA con 3′RARAx2)[93/100]	ogm[GRCh38]46,XY,**13q21.32q34(65889491_114352102)x2~3,**t(15;17)(q24.1;q21.2)(74029809;40335716)	46,XY**,+13q** ^2^,t(15;17)(q24.1;q21.2)
19	46,XX,t(6;9)(p22;q34)[15]	nuc ish 6p22(DEKx3),9q34(NUP214x3)(DEK con NUP214x2)[85/100]	ogm[GRCh38]46,XX,t(6;9)(p22.3;q34.13)(18232692;131152428),**11p12(41618837_42297919)x2~3**	46,XX,t(6;9)(p22.3;q34.13),**dup(11)(p12)**
21	46~48,XX,der(9)del(9)(p21)del(9) (q22q33),+21,+21[cp15]/46,XX[5]	nuc ish 8q22(RUNX1T1x2),21q22(RUNX1x3~4)[70/100],9q34(ABL1x2),22q11(BCRx2)[97/100]	* not described, because of chromothripsis	48,XX,**cth(9)**,+21,+21
24	46,XX[25]	negative	ogm[GRCh38]46,XX,**2q36.3(229862838_229886192)ins,9p12p11.2(39560026_40813220)x2~3,9q21.11(66749836_67475278)x2~3**	46,XX,**ins(2)(q36.3),dup(9)(p12p11.2),dup(9)(q21.11)**
3	46,XX,t(6;11)(q26;q23)[10]/ 46,idem,**del(1)(q24q41)**[4]/46,XX[1]	nuc ish 11q23(MLLx2)(5′MLL sep 3′MLLx1)[84/100]	ogm[GRCh38]46,XX,**2q36.3q37.2(229708729_235034836)x1,t(2;6)(q31.3;q22.31)(124062016;180428380),t(4;8)(p15.33;q24.21)(180428380;124062017),**t(6;11)(q27;11q23.3)(167843969;118493942),**t(8;14)(q11.21;q32.33)(47525976;105869036),8p23.1q24.21(10764562_129368956)**x**1**	46,XX,**del(2)(q36.3q37.2),t(2;6)(q31.3;q22.31),t(4;8)(p15.33;q24.21)**,t(6;11)(q27;q23.3),**del(8)(p23.1q24.21),t(8;14)(q11.21;q32.33)**
5	46,XX,t(9;11)(p21,q23)[2]/ 46,idem,der(21)**t(8;21)(q21;p13)**[7]/ 46,XX[6]	nuc ish 8q22(RUNX1T1x3),21q22(RUNX1x2)[72/100],11q23(MLLx2)(5′MLL sep 3′MLLx1)[94/100]	ogm[GRCh38]46,XX,8q13.1q24.3(66259972_142032191)2~3,t(9p24.3;11q23.3)(21118146;118493942),**t(14q24.3;17q21.32)(73466593;49017556),14q24.3q32.33(73473834_106873282)x1~2,17q21.32q25.3(49017556_83246392)x2~3**	46,XX,+8q ^5^,t(9;11)(p24.3;q23.3),**t(14;17)(q24.3;q21.32),del(14)(q24.3q32.33),dup(17)(q21.32q25.3)**
6	48,XX,**del(6)(q16q25),der(7)t(7;13)** **(p11;p11),+der(8)t(1;8)(q21;p11)**,+10 [17]/46,XX[4]	nuc ish 8q22(RUNX1T1x3),21q22(RUNX1x2)[67/100]	ogm[GRCh38]47,XX,1q21.1q44(143278152_248943333)x2~3,**t(6;7)(q21;p15.2)(109354469;27165827)**,8q11.1q24.3(45972483_145076125)x2~3,10p15.3q26.3(18514_133785266)x2~3	47,XX,+1q,**t(6;7)(q21;p15.2),**+8q,+10
11	**47,XX,+21[2]/49,idem,+6,+20[5]/** **92,XXYY[1]**/46,XX[18]	nuc ish 3q26(EVIx4)[7/100],8q22(RUNX1T1x2),21q22(RUNX1x3)[37/100],11q23(MLLx4)[6/100],16q22(CBFBx4)[6/100]nuc ish 6q23(MYBx3)[19/100],20q12(D20S108x3)[16/100],21q22 (AML1x3)[21/100] 3	ogm[GRCh38]46,XX,**16p13.3q24.3(4331324_88868384)inv**	46,XX,**inv(16)(p13.3q24.3)**
14	48,XY,+6,+6[1]/48,idem,del(3)(q13q26),**add(11)(p14)**[23]/46,XY[1]	-	ogm[GRCh38]48,XY,3q13.12q25.31(107775421_167698608)x1,**3q25.31q26.1(156499437_167703086)inv**,6p25.3q27(76216_170739897)x3~4,**t(11;17)(p15.4;q24.2)(3733790;67956414)**	48,XY,del(3)(q13.12q25.31),**inv(3)(q25.31q26.1)**,+6,+6,**t(11;17)(p15.4;q24.2)**
22	45,XY,der(4)del(4)(p11p15)del(4) (q11q24),−7,**add(13)(p11)**, t(17;19)(q22;q13)[20]	nuc ish 8q22(RUNX1T1x2),21q22(RUNX1x2)[100/100]	ogm[GRCh38]46,XY,**1p36.13p35.2(14490385_32907733)**x**1**,4p12(44197510_49078708)x1,4q12q13.1(51826792_63679087)x1,**t(4;7)(p15.1;p21.3)(32503668;9394985),7q11.21q36.3(62995089_157624118)**x**1,t(8;12)(q24.13;12p13.2)(125189890;11672773),13q14.2q14.3(48278417_51484127)**x**1,15q15.1(40315717_41591804)**x**1~2,16p13.2p12.3(9863863_16429873)**x**1~2,**t(17;19)(q22;q13.32)(58275730;45095385),**22q12.2q12.3(28643629_34061240)**x**1~2**	46,XY,**del(1)(p36.13p35.2)**,del(4)(p12),del(4)(q12q13.1),**t(4;7)(p15.1;p21.3),del(7)(q11.21q36.3),t(8;12)(q24.13;p13.2),del(13)(q14.2q14.3),del(15)(q15.1),del(16)(p13.2p12.3),**t(17;19)(q22;q13.32),**del(22)(q12.2q12.3)**

^1^ adapted from ISCN microarray nomenclature. ^2^ CNV confidence 0.1. ^3^ additional FISH diagnostic due to discrepancies in aneuploidies between karyotyping and OGM. ^4^ subclonal del(7). ^5^ map of chromosome 21 was found as an insertion in chromosome 8 in Bionano Access Server. * very complex chromothriptic pattern of abberrations, for details see text chapter 3.1.3. The background colors of column 1 in this table indicate identical or divergent results of karyotyping and OGM and corresponds to the colors in Figure 1. For details see legend of Figure 1. Structural Variants detected by only one of the two methods are indicated in bold.

## Data Availability

The retrospective analysis made use of the data of the pediatric patients enrolled in the AML-BFM registry 2017 (Ethics Committee of the Medical Faculty of University Duisburg-Essen, protocol code 17-7462-BO, 8 December 2017) and was conducted according to the guidelines of the Declaration of Helsinki. The data sets generated and/or analyzed during the current study are not publicly available but are available from the corresponding author on reasonable request.

## Supplementary Materials

The following supporting information can be downloaded at: https://www.mdpi.com/article/10.3390/cancers14092058/s1, Figure S1: Copy number variation plot of case #1; Table S1: Rare translocation calls; Table S2: Affected genes in deletions and duplication; Table S3: Primers.
