# Supplementary material for: Optical Genome Mapping as a Diagnostic Tool in Pediatric Acute Myeloid Leukemia
Source: Cancers (Basel). 2022 Apr 19;14(9):2058. doi: 10.3390/cancers14092058 (PMC9102001; doi:10.3390/cancers14092058)
Supplement: Supplementary file 1 [file cancers-14-02058-s001.zip › cancers-1659970-supplementary/supplement figure.pptx]

## Slide 1
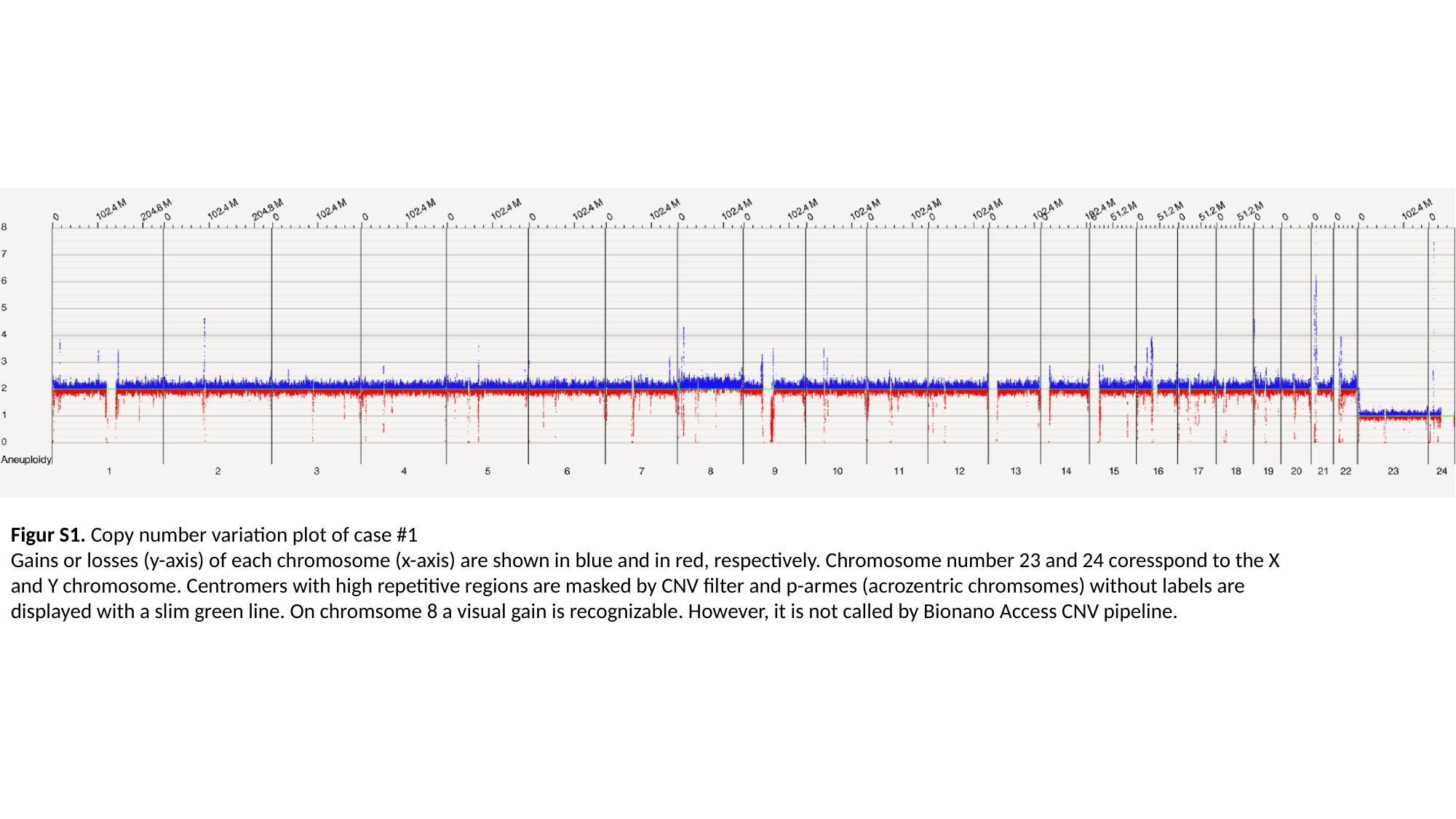

Figur S1. Copy number variation plot of case #1
Gains or losses (y-axis) of each chromosome (x-axis) are shown in blue and in red, respectively. Chromosome number 23 and 24 coresspond to the X and Y chromosome. Centromers with high repetitive regions are masked by CNV filter and p-armes (acrozentric chromsomes) without labels are displayed with a slim green line. On chromsome 8 a visual gain is recognizable. However, it is not called by Bionano Access CNV pipeline.
