# Supplementary material for: Optical Genome Mapping as a Diagnostic Tool in Pediatric Acute Myeloid Leukemia
Source: Cancers (Basel). 2022 Apr 19;14(9):2058. doi: 10.3390/cancers14092058 (PMC9102001; doi:10.3390/cancers14092058)
Supplement: Supplementary file 1 [file cancers-14-02058-s001.zip › cancers-1659970-supplementary/Table S1_rare translocation calls.pdf]

Table S1. Rare translocation calls

case #	translocation call
6	t(2;7)(q32.3;q21.11)
8	t(10;12)(p12.1;q14.1)
20	t(4;10)(q26;p12.1)
