# Supplementary material for: Optical Genome Mapping as a Diagnostic Tool in Pediatric Acute Myeloid Leukemia
Source: Cancers (Basel). 2022 Apr 19;14(9):2058. doi: 10.3390/cancers14092058 (PMC9102001; doi:10.3390/cancers14092058)
Supplement: Supplementary file 1 [file cancers-14-02058-s001.zip › cancers-1659970-supplementary/Table S3_Primers.pdf]

Table S3. Primers

Case	Name	Sequence (5‘->3‘)	Annealing temperature [°C]	Used for
#4	del(17)_FOR1	AGGGAAAACCCCAGATACCAAAC	60.7	primer walking
	del(17)_FOR2	CCCACCACTACTGCTATACCTC	62.1	primer walking, breakpoint spanning
	del(17)_FOR3	TGCATCTCTCTCAACTCAGACC	60.3	primer walking
	del(17)_REV1	TTGCCATTGATAGGGTAGGGC	59.8	primer walking, breakpoint spanning
	del(17)_REV2	TGCCTACAGTATATTAGGTGAAAGC	59.7	primer walking
	del(17)_REV3	TGTGGGAAGGGTTTTAGACGC	59.8	primer walking
#9	del(19)_FOR1	GACCTGACTCAAGCCCCTTC	61.4	primer walking, break point spanning
	del(19)_FOR2	GAGTGTTCTCAGGGCTTCCC	61.4	primer walking
	del(19)_REV1	CAGGACCCATCGACCACAAA	59.4	primer walking
	del(19)_REV2	GAGAGGTGGGAAGGAGGGTC	63.5	primer walking
	del(19)_REV3	GGGTGCCAAGAAAGGGAACT	59.4	break point spanning
#15	t(2;12)_FOR1	ATCAGCTTCACCTTGGGTCG	59.4	primer walking
	t(2;12)_FOR2	CCTATTCTGAGGCTGAATTATGGC	61.	break point spanning
	t(2;12)_REV1	CAGTGCCCTTCCATCCCATT	59.4	primer walking
	t(2;12)_REV2	ACAAGAGGCAACCCCATCAG	59.4	break point spanning
#22	t(8;12)_FOR1	TTGAGGGCAGTTGTCAAAGC	57.3	primer walking
	t(8;12)_FOR2	CTCCTCTCCCCTGCTCTCAT	61.4	primer walking
	t(8;12)_FOR3	TAATGAGAGACGGCAGCCG	58.8	primer walking/sequencing
	t(8;12)_FOR4	AAATTCCTTAAAATGCTCAACAA	51.7	primer walking/sequencing
	t(8;12)_FOR5	ATACAGGTCATAGGCATGCTTTGT	59.3	primer walking/sequencing
	t(8;12)_FOR6	CCAATTCTTGAAAACACTTTTGTGTG	58.5	primer walking/sequencing
	t(8;12)_FOR7	TGCACATACATCATCTCGCGT	57.90	break point spanning
	t(8;12)_REV1	CCTTTCCTGCGTGTGGATGT	59.4	primer walking
	t(8;12)_REV2	TCTCCAAAGCCATCAGCCAG	59.4	primer walking
	t(8;12)_REV3	AATGTACAATATGTGCTAACT	50.6	primer walking/sequencing
	t(8;12)_REV4	GAGGGCAGGGACTCTATGAC	61.40	primer walking/sequencing
	t(8;12)_REV5	CCTCAGATACCTGGGCAGC	60.99	primer walking/sequencing
	t(8;12)_REV6	CACCCCATTAACGGAGGACA	59.40	break point spanning
